# MicroRNA-219c-5p regulates bladder fibrosis by targeting FN1

**DOI:** 10.1186/s12894-020-00765-5

**Published:** 2020-12-07

**Authors:** Bowen Liu, Yafei Ding, Peng Li, Tao Wang, Siyuan He, Zhankui Jia, Jinjian Yang

**Affiliations:** 1 Department of Urology, Zhengzhou University First Affiliated Hospital, Zhengzhou, 450052 China; 2 Zhengzhou Institute of Urology, Zhengzhou University First Affiliated Hospital, Zhengzhou, 450052 China

**Keywords:** Multiple sclerosis, Experimental autoimmune encephalomyelitis, Bladder fibrosis, Fibronectin-1, MicroRNA-219c-5p

## Abstract

**Background:**

We found that the bladders of multiple sclerosis mice were significantly fibrotic. This study aimed to investigate the relationship between fibronectin 1 (FN1) and bladder fibrosis, as well as the microRNAs involved in FN1 regulation.

**Methods:**

The degree of bladder smooth muscle fibrosis was observed by immunohistochemistry. In addition, we used quantitative real-time polymerase chain reaction (RT-qPCR) and Western blotting to determine FN1 expression in bladders with different grades of fibrosis. Bioinformatics analysis revealed that miR-199a-3p, miR-219c-5p and miR-3572-3p could inhibit FN1 synthesis. Therefore, miR-199a-3p, miR-219c-5p and miR-3572-3p were overexpressed or knocked down in bladder smooth muscle cells (BSMCs), and the respective transfection and FN1 knockdown efficiencies were detected by RT-qPCR. Only miR-219c-5p overexpression and knockdown produced the expected results. A dual luciferase reporter assay was used to determine the targeting relationship between miR-219c-5p and FN1. Flow cytometry and Cell Counting Kit 8 (CCK8) experiments confirmed that miR-219c-5p reduced FN1 expression and affected the biological activity of smooth muscle cells. Agomir and anagomir of miR-219c-5p were transfected in vivo to observe the change of bladder fibrosis in mice.

**Results:**

With increasing bladder fibrosis, FN1 expression increased, while miR-199a-3p, miR-219c-5p, and miR-3572-3p expression levels decreased. The RT-qPCR results after transfection showed that only miR-219c-5p could regulate FN1. Indeed, the dual luciferase reporter assay results indicated that miR-219c-5p targeted FN1 directly. CCK8 and cell cycle assays showed that miR-219c-5p overexpression inhibited BSMC proliferation, while miR-219c-5p knockdown promoted BSMC proliferation. An apoptosis assay showed that miR-219c-5p overexpression promoted apoptosis, while miR-219c-5p knockdown inhibited BSMC apoptosis. The agomir and anagomir transfected with miR-219c-5p in vivo found that the bladder fibrosis of the mice in the agomir group was reduced, and the anagomir group was worse.

**Conclusions:**

Our findings indicate that FN1 up-regulation and miR-219c-5p down-regulation play an important role in the development of bladder fibrosis, and miR-219c-5p participates in bladder fibrosis by regulating FN1 expression. Thus, a novel antifibrotic function of miR-219c-5p is proposed, which may represent a potential target for the diagnosis and treatment of bladder fibrosis.

## Background

Multiple sclerosis (MS) is a diffuse inflammatory autoimmune disease characterized by multiple symptoms and signs [1, 2]. It is currently believed that MS patients suffer mainly from myelin-specific T cell-mediated autoimmune demyelination diseases. Demyelination of the urinary reflex nerve leads to inconsistent bladder detrusor and sphincter movement. Therefore, MS patients often suffer frequent urination, urgency and urinary retention [3–5]. Although many reports have investigated the possible mechanisms of lower urinary tract symptom development in MS, the mechanism of bladder fibrosis as the ultimate outcome of bladder dysfunction has not been clearly elucidated [6, 7]. The experimental autoimmune encephalomyelitis (EAE) model is currently the most commonly used animal model of MS. The myelin autoantigen specifically activates helper T cells in the brain, causing inflammatory infiltration of the central nervous system and loss of myelin. The biochemical, immunological and pathological features of EAE are very similar to those of MS, so this model is widely used to assess the etiology of MS and to find different treatment options [8–10]. PLP139 sensitized SJL/J mice established a relapsing and relieving MS model, while MOG35-55 immunized C57BL/6J mice established an irreversible chronic progressive MS model, where demyelination and axonal damage were obvious. Considering the long-term chronic progression of bladder fibrosis, the C57BL/6J mouse model was chosen. Bladder fibrosis is the ultimate outcome of bladder dysfunction [11, 12]. Fibrosis is described as the "wrong wound healing process", and while many mechanisms may be involved in its development, they are all based on BSMCs activation and the resulting extracellular matrix (ECM) components [13]. Wang et al. [14] reported for the first time that hypoxia induced microRNA-101b and inhibited the TGF-β signaling pathway, thereby inhibiting fibrosis in BSMC. Altuntas et al. [7] were the first to report that bladder fibrosis and bladder remodeling corresponding to the severity of EAE may be due to increased CTGF expression and increased connective tissue growth. Antifibrotic therapy may be an effective way to control bladder dysfunction, but it has not been well studied [15–17].

Fibronectin is an extracellular macromolecular membrane protein present on the surface of animal cells and is the main noncollagen glycoprotein in the ECM and basement membrane. It is also very abundant in the ECM of various tumors, including osteosarcoma, leiomyosarcoma, and gastric cancer [18]. Fibronectin 1 (FN1), which belongs to the FN family, is involved in a variety of cellular biological processes and plays a role in diseases such as fibrosis [19]. Wei Wang et al. [20] first reported that lncRNA GAS5 can adsorb miR-96-5p, reduce the inhibition of miR-96-5p on fibrosis protein FN1, and then aggravate renal fibrosis. We found that the more severe the bladder fibrosis is, the higher the FN1 levels are. We speculate that by inhibiting FN1 synthesis, the process of fibrosis in bladder smooth muscle cells (BSMCs) can be reduced.

A large number of studies have shown that miRNA is involved in a variety of cell biological processes such as cell proliferation and apoptosis. Therefore, miRNAs may be critical for the diagnosis and treatment of certain diseases [21]. Many miRNAs have been found in the brain and spinal cord tissue of EAE mice as biomarkers for MS. Ilona B. Bruinsma et al. [22] reported that detecting the loss of miR-219 in patient cerebrospinal fluid may be a candidate biomarker for MS diagnosis. MicroRNAs (miRNAs) are short-chain noncoding RNAs of approximately 22 nucleotides in length whose primary function is to participate in biological processes by inducing mRNA degradation. Many miRNAs, including miR-26b-5p and miR-200c, are involved in the development of fibrosis [23, 24]. Notably, regulating the expression of these miRNAs can prevent fibrogenesis in vitro and in vivo, suggesting the importance of miRNAs as potential targets for the treatment of fibrotic diseases [25]. In this study, we used miRDB (http://mirdb.org/) to predict potentially targeted miRNAs for FN1. After comparative analysis, miR-199a-3p, miR-219c-5p and miR-3572-3p were all predicted to target FN1.

The bladder wall is composed mainly of a mucosal layer, a submucosa layer, a muscle layer and perimuscular connective tissue. Bladder fibrosis is caused mainly by the pathological proliferation of BSMCs in the muscular layer of the bladder wall. A considerable amount of matrix deposition in the cytoplasm of BSMCs affects bladder detrusor compliance and leads to bladder remodeling and fibrosis. Therefore, BSMCs were used as an experimental model in our study. To investigate whether microRNAs can inhibit FN1 synthesis and block the progression of BSMC fibrosis, we overexpressed and knocked down microRNAs in BSMCs in vitro. The RT-qPCR results after transfection showed that miR-219c-5p was able to regulate FN1.

In this study, we established an EAE model to simulate bladder fibrosis in MS patients and found that bladder fibrosis was directly proportional to FN1. Through in vitro and vivo experiments, we found that miR-219c-5p could inhibit the progression of bladder smooth muscle fibrosis by targeting FN1. This study aimed to investigate the significance of miR-219c-5p expression in bladder fibrosis in vitro and in vivo. In addition, this study determined that miR-219c-5p may target the FN1 gene directly, and the miR-219c-5p/FN1 signaling pathway may be useful in the diagnosis and treatment of bladder fibrosis.

## Methods

### Animals

80 female C57BL/6 J mice (4–9 weeks old) were purchased from Charles River. All mice were kept in a specific pathogen-free (SPF) animal room at the Zhengzhou University Animal Experimental Center and exposed to a 12:12 h light–dark cycle (light from 6 am to 6 pm).

### EAE model

To induce chronic EAE, 40 animals were injected subcutaneously with an emulsion of MOG 35–55 in complete Freund’s adjuvant (CFA) (Sigma, USA). Then, pertussis toxin (Sigma, USA; 0.2 μg per animal) in PBS was administered on days 0, 3, and 7 after immunization [7]. The mice were scored daily according to the following signs of nerve damage: 0, no disease; (1) tail weakness and/or moderately awkward gait and/or poor righting ability; (2) tail weakness and/or moderately awkward gait; (3) hind limb paralysis or mild forelimb weakness (or both); (4) limb paralysis; and (5) quadriplegia with detained urine or death. Another group of animals was injected subcutaneously with CFA emulsion and used as the normal control group. Forty mice were scored daily for signs of neurological impairment according to clinical symptom (CS) score criteria up to 50 days after induction. Animals received standard Purina chow and had free access to water. On the 50th day after immunization, the mice were placed in transparent glassware, and excess isoflurane was added. When the mouse does not have spontaneous breathing and blinking reflexes, the follow-up operation can be performed. The experimental protocol was approved by the Ethics Committee of the First Affiliated Hospital of Zhengzhou University and confirmed with the Guide for the Care and Use of Laboratory Animals.

In order to analyze the effect of miR-219c-5p in mice on bladder fibrosis in EAE mice, another 40 mice were used to establish an EAE mouse model. On the 30th day, randomly selected CS3 mice were divided into EAE-miR-219c-5p agomir group, EAE-miR-219c-5p antagomir group and EAE-NC group, 3 mice in each group. MiR-219c-5p agomir (10 nmol), miR-219c-5p antagomir (10 nmol) and PBS were injected into the tail vein once a week. On the 50th day, the mice were killed by cervical dislocation after anesthesia. The bladder tissues of the mice were taken for HE and Masson`s trichrome staining. The degree of fibrosis and the expression of FN1 were observed by FN1 immunohistochemistry.

### Prediction of potentially targeted miRNAs that inhibit FN1

The miRDB (http://mirdb.org/) and miRBase (http://www.mirbase.org) databases were used to predict FN1-targeting microRNAs. After comparing the context score, context score percentile, weighted context score, and conserved branch length, miR-199a-3p, miR-219c-5p and miR-3572-3p were predicted to interact with FN1.

### Mouse BSMC primary culture

BSMCs were purchased from Otwo Biotech (HTX3129; Shenzhen, China) and verified by a-SMA immunofluorescence. The BSMCs were cultured in Dulbecco's modified Eagle’s medium—high glucose (DMEM-H; Gibco, Thermo Fisher Scientific, Waltham, MA) containing 10% fetal bovine serum (FBS) (Gibco, Thermo Fisher Scientific) and 100 U/ml cyan-streptomycin. Anti-α-SMA (MA1-06110; Thermo Fisher Scientific; 1:200) and goat anti-rabbit IgG H&L (Alexa Fluor488) (ab150077; Abcam, USA; 1:1000) immunofluorescence staining was used to identify BSMCs. Only cells at passage numbers 3 to 10 were used for all experiments.

### Overexpression or knockdown of miRNAs in BSMCs

One day before transfection, cells were starved by seeding in medium supplemented with 2% FBS at a density of 5 × 10^4^ cells/ml. On the day of transfection, the medium was changed to 10% FBS medium, and the cells were transfected with miRNA mimic, NC mimic, miRNA inhibitor or NC inhibitor (RiboBio, Guangzhou, China) using a riboFECT™ CP Transfection Kit (RiboBio, Guangzhou, China) according to the manufacturer’s instructions. The miRNA mimic and inhibitor used are listed in Table 1.

**Table 1:** List of gene

Gene name	Sequence
miR-199a-3p mimics	5′-AUUGGUUACACGUCUGAUGACA-3′
miR-199a-3 inhibitor	5′-UAACCAAUGUGCAGACUACUGU-3′
miR-219c-5p mimics	5′-GGACGUCCAGACGCAACUCUCG-3′
miR-219c-5p inhibitor	5′-CGAGAGUUGCGUCUGGACGUCC-3′
miR-3572-3p mimics	5′-UACACUUGUCCUUCUUUCCCCAG-3′
miR-3572-3p inhibitor	5′-CUGGGGAAAGAAGGACAAGUGUA-3′
mimics NC	5′-UUUGUACUACACAAAAGUACUG-3′
inhibitor NC	5′-UUUGUACUACACAAAAGUACUG-3′

### miR‑219c-5p target prediction and luciferase reporter assay

Potential miR-219c-5p binding sites were predicted using online database programs with different algorithms, including miRDB (http://mirdb.org/) and miRBase (http://www.mirbase.org). For the luciferase assay, BSMCs (3 × 10^3^ cells/well) were first seeded in 96-well plates. Then, 1 day later, luciferase reporter plasmids [pmiR-FN1-wild type (WT) or pmiR-FN1-mutant (Mut) (RiboBio, Guangzhou, China)] were cotransfected with miR-219c-5p mimic or NC mimic into BSMCs. Luciferase activity was determined 48 h after transfection using a GLOMAX 96 spectrophotometer (Promega Corporation) following the manufacturer's protocol. Firefly luciferase activity was normalized to Renilla luciferase activity.

### Quantitative real-time polymerase chain reaction

Total RNA was isolated from bladder tissue or BSMCs using TRIzol (Leagene Biotechnology, Beijing, China) according to the manufacturer's protocol. For miRNA RT-qPCR, reverse transcription was performed using ReverTra Ace qPCR RT Master Mix (FSQ-101; TOYOBO, Japan). For FN1 RT-PCR, reverse transcription was performed using ReverTra Ace qPCR RT Master Mix and gDNA Remover (FSQ-301; TOYOBO, Japan). As described above, cDNA obtained by reverse transcription was used for real-time PCR. qPCR was performed using a SYBR Green Mix (Applied Biosystems; Roche, Inc.) in a 20-μl reaction at 95 °C for 2 s, 60 °C for 20 s, and 70 °C for 10 s (40 cycles). Relative miRNA expression levels and other indicators were calculated in triplicate using the 2^−ΔΔCT^ method. Expression levels were normalized to β-actin or U6 levels, and the primers used are listed in Table 2.

**Table 2:** List of primers

Gene name	Primer sequences
β-actin	F-AAATGTGGCTGAGGACTTTGTAC
	R-GGACTTCCTGTAACCACTTATTTCA
U6	F-GCTCGCTTCGGCAGCACA
	R-GAGGTATTCGCACCAGAGGA
FN1	F-ACCCGTTTTCATCCAACAAGAG
	R-CGGTATCCAGACACCACACTATCA

*FN1* Fibronectin 1, *F* forward, *R* reverse

### Hematoxylin and eosin and Masson’s trichrome staining

Mouse bladders were soaked overnight in 4% paraformaldehyde. The tissues were cut into 5-μm paraffin sections for bladder fibrosis analysis. According to the protocol, the paraffin sections were stained with hematoxylin and eosin (H&E) and Masson’s trichrome. The amount of collagen, the number of smooth muscle cells, and the ratio of collagen to smooth muscle cells were calculated by using an image analysis system (ImageJ 1.80). The ratio of collagen to smooth muscle cells was used to quantify the degree of fibrosis in the bladder tissue.

### Immunohistochemistry

Immunohistochemical (IHC) staining for streptavidin-peroxidase (SP) was used to detect FN1 expression in bladder tissue. After sectioning, conventional dewaxing, gradient ethanol hydration, and antigen retrieval were performed in sequence. Subsequently, the sections were incubated with 3% hydrogen peroxide for 20 min, blocked with serum for 10 min, and incubated with the primary antibody rabbit anti-mouse FN1 (ab45688; Abcam, USA; 1:200) overnight at 4 °C. The sections were then incubated with horseradish peroxidase-labeled goat anti-rabbit secondary antibody (ab205718; Abcam, USA; 1:2000) for 30 min and stained with DAB (Sigma, USA). After counterstaining with hematoxylin, all slides were hydrated, clarified, fixed and observed, and the positive cell index was determined.

### Cell proliferation assay

Cell proliferation was tested using a Cell Counting Kit 8 (CCK8) (CK04; DOJINDO, Japan; 10 µl) assay. The BSMCs to be transfected were inoculated into 96-well plates. Each well was inoculated with approximately 1000 cells, and 6 replicate wells were assayed for each group. After 24, 48, 72 and 96 h of transfection, 10 μl of CCK8 reagent was added to each well and incubated for 1 h. Sample absorbance was read at 450 nm on a microplate reader (SpectraMax-M5, Sunnyvale, CA).

### Flow cytometry

After 48 h of transfection, cells were treated with 0.25% trypsin and harvested. Approximately 1 × 10^5^ cells were counted, fixed with 75% cold ethanol (Solarbio Life Sciences, Shanghai, China) for 4 h, and then treated with 0.5% Triton-X100 for 10 min. Triton-X100 was removed by centrifugation, and the cells were stained with 200 μl propidium iodide (PI) (BD, USA) at room temperature for 30 min in the dark. A flow cytometer (ACCURI C6 PLUS, BD, USA) was used to analyze the cell cycle, and the experiments were repeated three times. Similarly, cells were treated with 0.25% trypsin without ethylenediaminetetraacetic acid. Approximately 1 × 10^5^ cells were counted, and 10 μl Annexin V-fluorescein isothiocyanate (FITC) (BD, USA) and PI (BD, USA) each was added and incubated at room temperature for 15 min in the dark. The rate of apoptosis (percentage of apoptotic cells relative to the total number of cells) was estimated by flow cytometry. Representative results of three independent experiments are shown.

### Western blot assay

An equal amount of each protein sample (20 μg) was separated using 12% sodium dodecyl sulfate polyacrylamide gel electrophoresis (SDS-PAGE) at 120 V. The proteins were transferred to a polypropylene difluoride (PVDF) membrane at a constant flow of 400 mA for 5 h under ice bath conditions. The PVDF membrane was incubated for 30 min at room temperature in 5% skim milk to block nonspecific banding. Then, the PVDF membrane was incubated with rabbit anti-mouse-FN1 (ab45688; Abcam, USA; 1:10,000) or anti-β-actin (AC026; ABclonal, Wuhan, China; 1:10,000) primary antibody at 4 °C overnight. After washing once with TBS and 3 times with TBST, the PVDF membrane was incubated with horseradish peroxidase-conjugated goat anti-rabbit IgG secondary antibody (AS003; ABclonal, Wuhan, China; 1:10,000) for 1 h at room temperature. The PVDF membrane was washed 5 times with TBST, and the proteins of interest were detected using an enhanced chemiluminescence (ECL) system. Densitometry analysis of the protein bands was performed using ImageJ software (National Institutes of Health, Bethesda, MA, USA) and normalized to the gray value of β-actin. This experiment was replicated three times.

### Statistical analysis

All data were obtained from three independent replicates and are shown as the mean ± standard deviation. Comparisons between two sets of samples were performed using Student's t test. One-way ANOVA or two-way ANOVA was used for comparisons between single-factor or two-factor multiple groups. *P* < 0.05 was chosen to represent a statistically significant difference. Statistical analysis was performed using SPSS software (SPSS for Windows 21.0; SPSS Inc., Chicago, IL, USA).

## Results

### EAE induced bladder wall fibrosis in mice

**Fig. 1:**
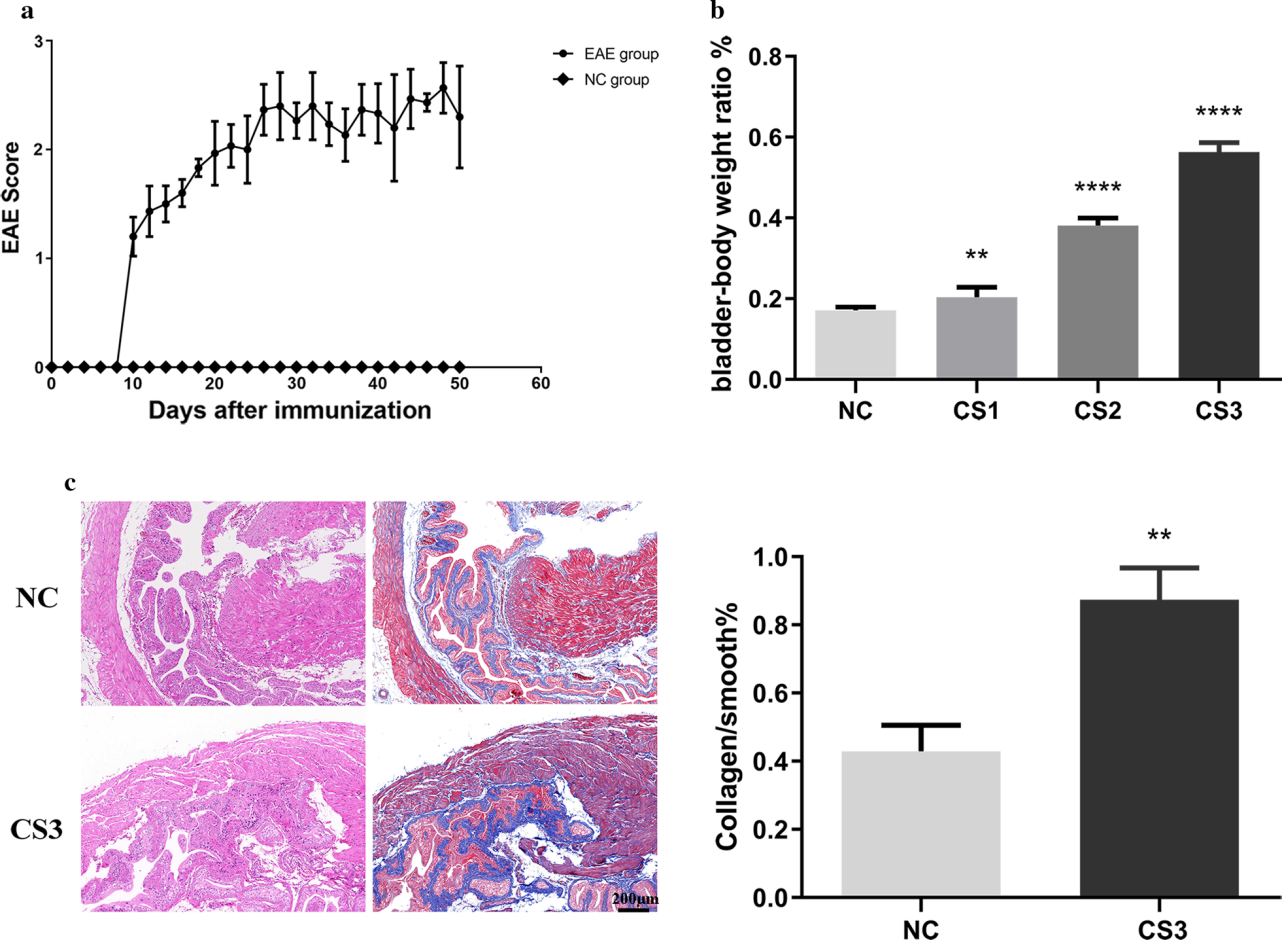
EAE-induced bladder wall fibrosis in mice. Female C57BL/6 mice were used to establish EAE using MOG 35–55. After immunization, the disease development was scored according to clinical symptoms (CS), and the body weight and neurological deficit of the mice were examined daily and scored on a scale of 0–5, where CS0 = no disease; CS1 = tail weakness and/or moderately awkward gait and/or poor righting ability; CS2 = tail weakness and/or moderately awkward gait; CS3 = hind limb paralysis or mild forelimb weakness (or both); CS4 = limb paralysis; and CS5 = quadriplegia with detained urine or death. **a** EAE progression in mice is indicated by the mean clinical score. The error bars show ± SE. **b** The bladder-to-body weight ratios were significantly greater in EAE mice than in normal control mice (NC) (n = 3 mice/group). **c** Only the degree of bladder smooth muscle layer tissue fibrosis was evaluated. Left: H&E, Right: Masson’s trichrome stain. The red areas indicate smooth muscle, and the blue areas indicate connective tissue. The collagen-smooth-muscle ratio was determined by the mean Masson’s trichrome staining data in the NC and CS3 groups (n = 3 mice/group). The data are expressed as the mean ± SD. NC, normal control group; CS, clinical symptoms. **P < 0.01, ****P < 0.0001 versus control. Scale bar = 200 μm

On the 10th day after immunization, EAE mice were observed to show signs of neurological deficits, while the normal control mice (immunized with CFA alone) were normal. We found that the symptoms of EAE mice were not persistent but rather presented a cycle of sustained remission-relapse. However, mice in the remission phase were not completely normal and still had tail weakness. Figure 1a shows the mean CS score of mice in the EAE model group from day 0 to day 50. The control group (NC) did not show any clinical symptoms throughout the time period. Fifty days after immunization, the mice were scored by CS, including 10 in the NC group, 7 in the CS1 group, 9 in the CS2 group, 12 in the CS3 group, and 2 in the CS4 group. At 50 days postimmunization, the bladder-body weight ratio was significantly higher for the EAE model group (CS1/2/3 group) than for the NC group (Fig. 1b , *P* < 0.0001), whereas the mean body weight was not significantly different between NC mice and EAE mice (Table 3, *P* > 0.05).

**Table 3:** Bladder weight, FN1 and miR-219c-5p expression in mice with different EAE scores

Variable	EAE score				F Value	*P* Value
	0	1	2	3		
Body weight/g	27.99 ± 0.56	27.91 ± 0.78	25.81 ± 1.05	25.68 ± 0.87	0.5882	0.646
Bladder weight to total body weight (%)	0.17 ± 0.01	0.20 ± 0.02	0.38 ± 0.02	0.56 ± 0.02	1002	< 0.0001
FN1	1.00 ± 0.10	1.23 ± 0.70	1.59 ± 0.14	3.01 ± 0.10	342.7	< 0.0001
miR-199a-3p	3.40 ± 0.53	2.73 ± 0.25	1.93 ± 0.40	1.03 ± 0.15	23.6	< 0.0001
miR-219c-5p	3.10 ± 0.10	1.73 ± 0.08	1.23 ± 0.06	1.00 ± 0.10	376.4	< 0.0001
miR-3572-3p	4.50 ± 0.50	3.53 ± 0.55	2.03 ± 0.25	1.03 ± 0.15	44.6	< 0.0002

Values are means ± SE; n = 3 mice/group. *P* < 0.05 by ANOVA among four groups; expression levels were normalized to β-actin or U6

To further determine whether prolonged urinary retention of the neurogenic bladder can lead to bladder wall fibrosis, we performed histological and morphological analyses by H&E and Masson’s trichrome staining. Only the degree of bladder smooth muscle layer tissue fibrosis was evaluated. H&E and Masson’s staining showed that the CS3 group was significantly more fibrous than the NC group (Fig. 1c, *P* < 0.01). Only the degree of bladder smooth muscle layer tissue fibrosis was evaluated. These results suggest that the higher the CS score of EAE mice is, the more severe the bladder fibrosis is.

### FN1 expression in EAE-induced fibrotic bladder tissue

**Fig. 2:**
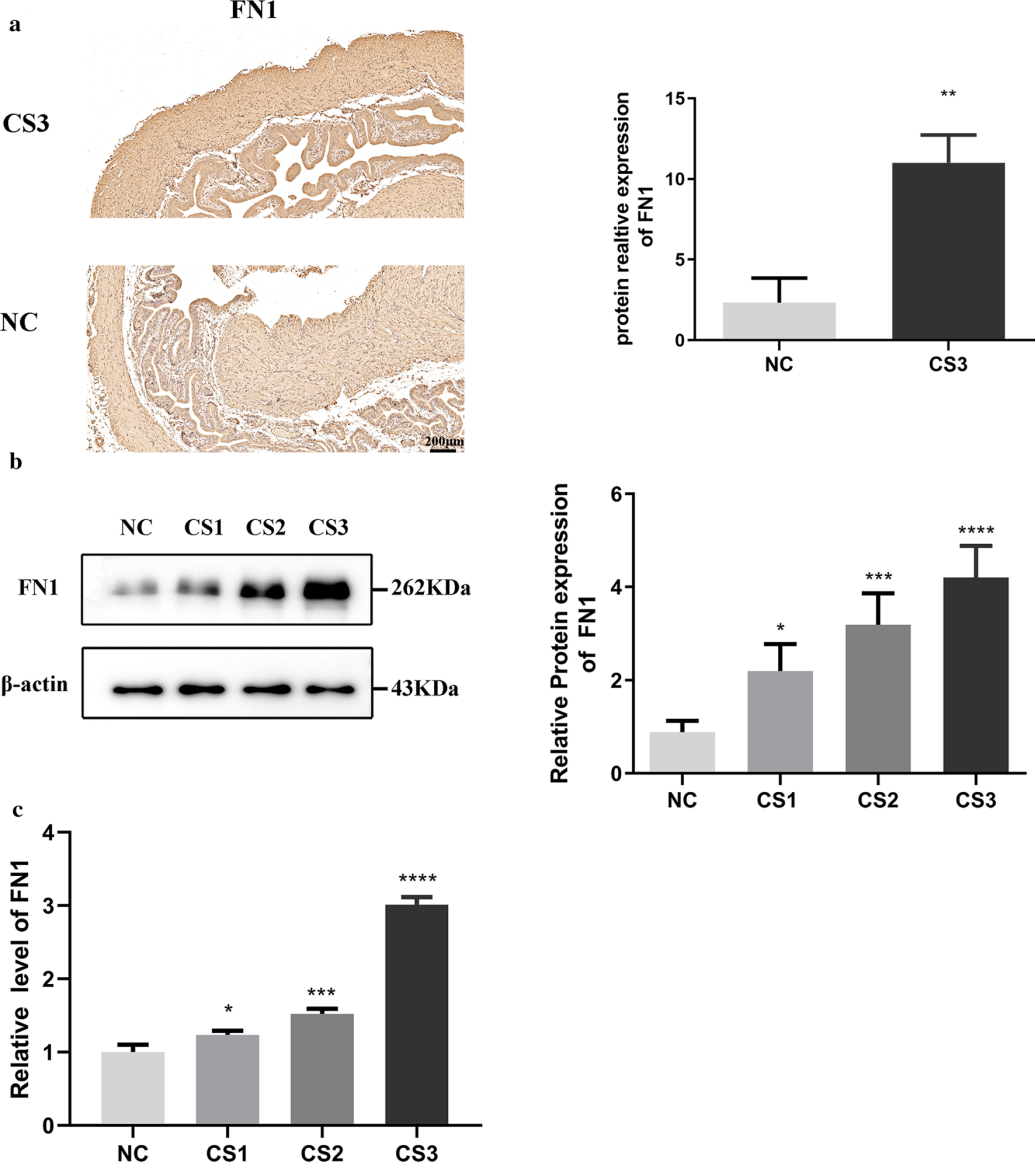
FN1 expression in EAE-induced fibrotic bladder tissue. **a** The expression of FN1 in bladder fibrosis with different scores was analyzed by immunohistochemistry. **b** The expression of FN1 in bladder fibrosis with different scores was analyzed by Western blotting. **c** The expression of FN1 in bladder fibrosis with different scores was analyzed by RT-qPCR. The findings were from three separate experiments. The data are expressed as the mean ± SD. NC, normal control group; CS, clinical symptoms; FN1, fibronectin 1. ^ns^*P* > 0.05, *P < 0.05, **P < 0.01, ***P < 0.001, ****P < 0.0001 versus control. Scale bar = 200 μm

We found a potential correlation between bladder fibrosis and FN1 by detecting protein and mRNA expression levels in bladders with varying degrees of fibrosis. As the clinical symptoms of EAE model mice deteriorated, the FN1 expression level increased significantly. We detected an increase in FN1 expression by immunohistochemistry (Fig. 2a, *P* < 0.01), Western blot analysis (Fig. 2b, *P* < 0.0001) and RT-qPCR (Fig. 2c, *P* < 0.0001) as bladder fibrosis was aggravated. These results indicate that the more severe the fibrosis of the bladder tissue is, the higher the FN1 content of the bladder tissue will be. In other words, the degree of bladder tissue fibrosis is positively correlated with the expression of FN1.

### Prediction of miRNAs targeting FN1 and detection of miRNA expression in bladders with different degrees of fibrosis

**Fig. 3:**
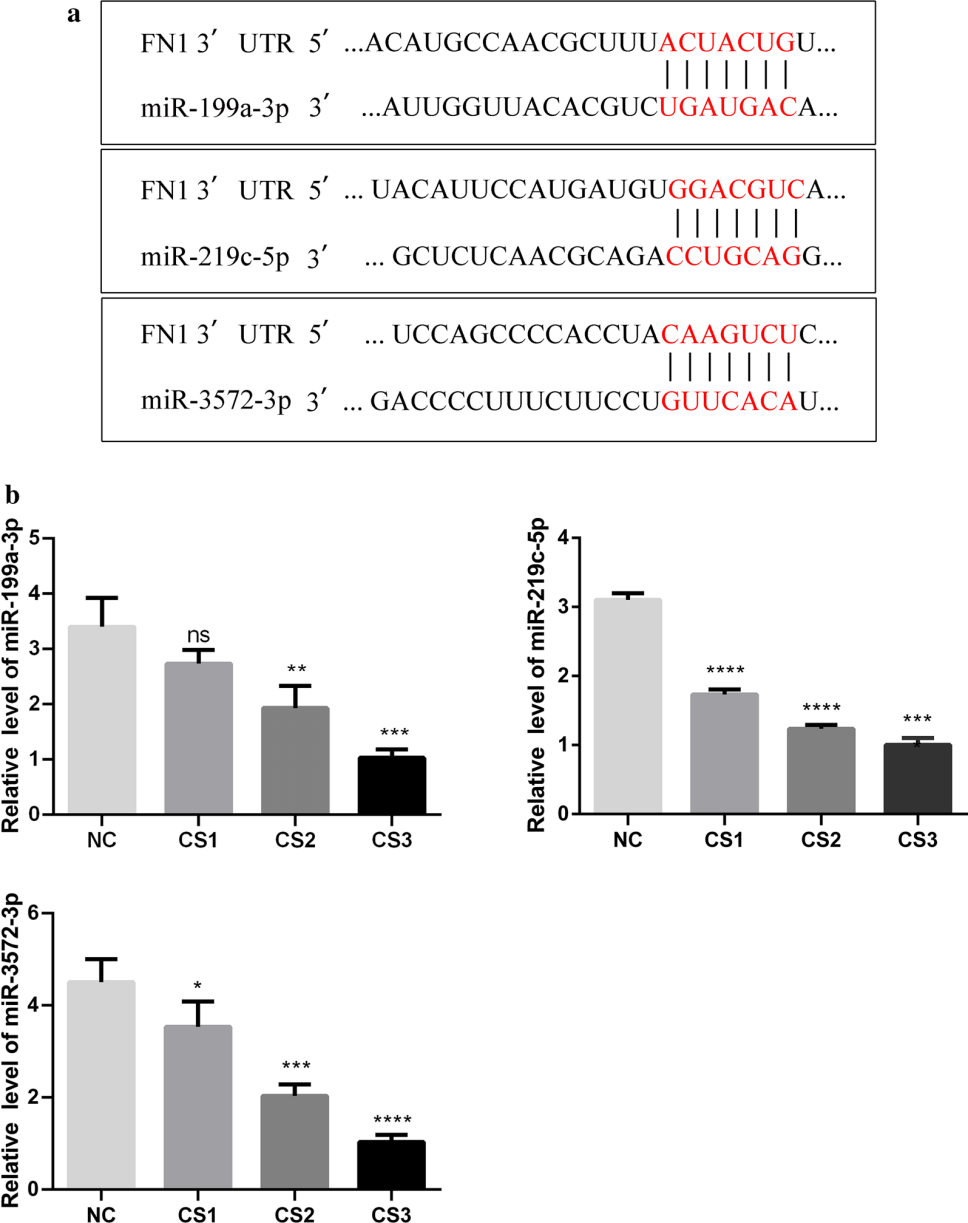
Prediction of miRNAs targeting FN1 and detection of miRNA expression in bladders with different degrees of fibrosis. **a** Database prediction that miR-199a-3p, miR-219c-5p, and miR-3572-3p target FN1. **b**–**d** The expression levels of miR-199a-3p, miR-219c-5p, and miR-3572-3p in different fibrotic bladders were detected by RT-qPCR. The findings were from three separate experiments. The data are expressed as the mean ± SD. NC, normal control group; CS, clinical symptoms; FN1, fibronectin 1. ^ns^*P* > 0.05, *P < 0.05, **P < 0.01, ***P < 0.001, ****P < 0.0001 versus control

The miRDB (http://mirdb.org/) and miRBase (http://www.mirbase.org) databases were used to predict FN1-targeting microRNAs. After comparing the context score, context score percentile, weighted context score, and conserved branch length, it was found that miR-199a-3p, miR-219c-5p and miR-3572-3p could interact with FN1 (Fig. 3a). Next, the miRNA expression levels in the bladder with different scores were detected by RT-qPCR (Fig. 3b–d). We found that the miR-199a-3p, miR-219c-5p and miR-3572-3p expression levels decreased with increasing bladder fibrosis, which was the opposite of the FN1 expression trend. These results indicate that the high FN1 expression during bladder fibrosis may potentially be related to the reductions in these miRNAs.

### Identification of BSMCs and miRNA transfection

**Fig. 4:**
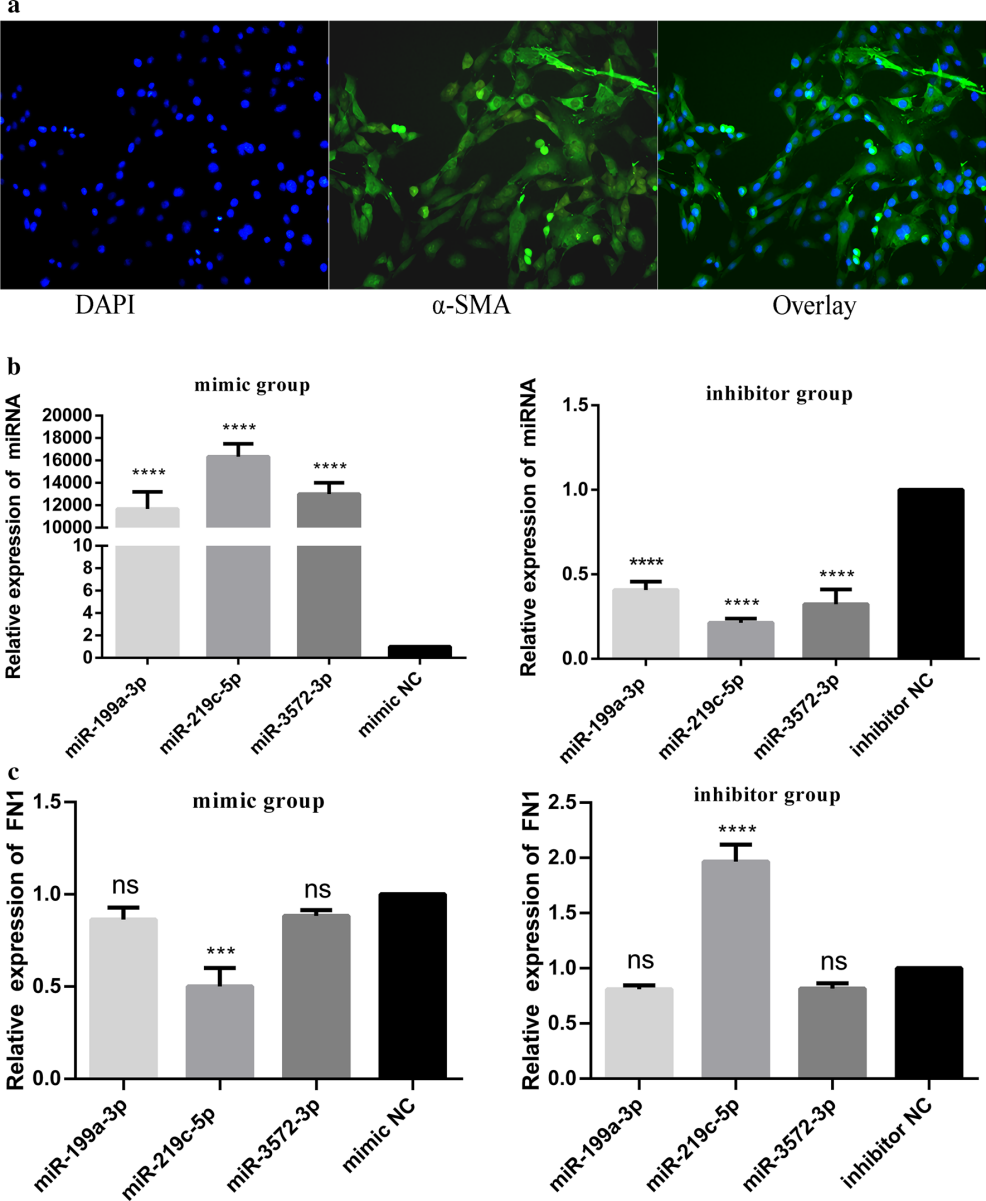
Identification of BSMCs and miRNA transfection. **a** Under a fluorescence microscope, almost all cells were stained green, indicating the cytoskeleton, and blue, indicating the nucleus (magnification: × 200). **b** The transfection efficiencies of miR-199a-3p, miR-219c-5p and miR-3572-3p were detected by RT-qPCR. **b** The effect of miR-199a-3p, miR-219c-5p and miR-3572-3p on FN1 was detected by RT-qPCR. The findings were from three separate experiments. The data are expressed as the means ± SD. CS, clinical symptoms; FN1, fibronectin 1. ^ns^*P* > 0.05, *P < 0.05, **P < 0.01, ***P < 0.001, ****P < 0.0001 versus control

Since the main component of the bladder wall is BSMCs, we cultured BSMCs in vitro for experiments. BSMCs were identified by immunofluorescence staining for α-SMA (Fig. 4a). The transfection efficiencies of miR-199a-3p, miR-219c-5p and miR-3572-3p were detected by RT-qPCR (Fig. 4b). Then, the effect of miR-199a-3p, miR-219c-5p and miR-3572-3p on FN1 was detected by RT-qPCR (Fig. 4c). We found that only miR-219c-5p produced the expected results when the transfection efficiencies among miR-199a-3p, miR-219c-5p and miR-3572-3p were the same. That is, FN1 was decreased (*P* < 0.001) when miR-219c-5p was overexpressed, and FN1 was increased (*P* < 0.0001) when miR-219c-5p was knocked down. In summary, miR-219c-5p is most likely one of the miRNAs targeting FN1.

### miR-219c-5p binding to FN1

**Fig. 5:**
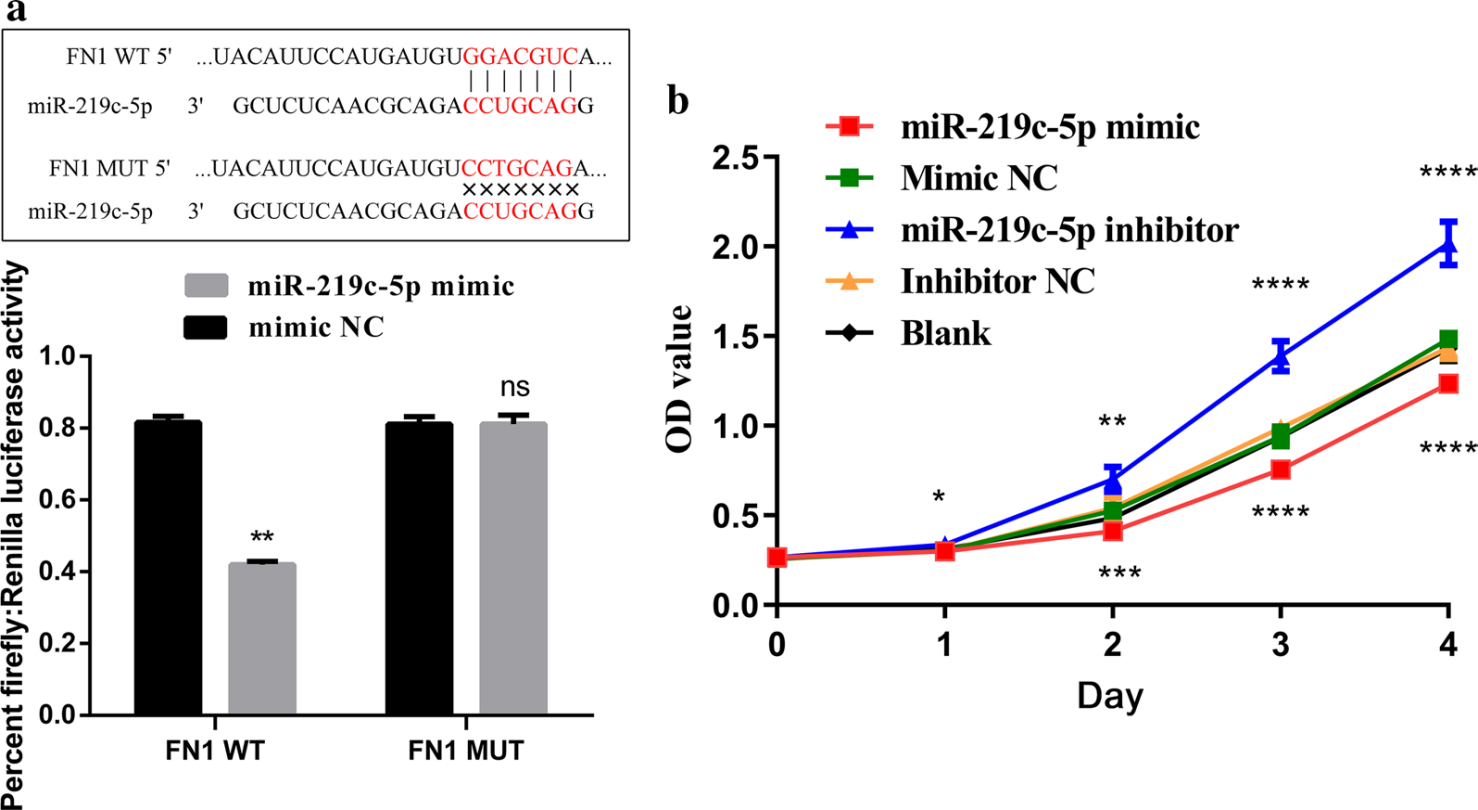
miR-219c-5p targets FN1 and affects BSMC proliferation. **a** miR-219c-5p and FN1 3′-UTR binding and luciferase activity detection in BSMCs (n = 3). **b** The OD_450_ values of the cells were measured via CCK8 assay. The findings were from three separate experiments. The data are expressed as the mean ± SD. WT, wild type; MUT, mutant; FN1, fibronectin 1; 3′-UTR, three prime untranslated region. ^ns^*P* > 0.05, *P < 0.05, **P < 0.01, ***P < 0.001, ****P < 0.0001 versus mimic NC or inhibitor NC

Bioinformation prediction websites such as miRDB (http://mirdb.org/) and miRBase (http://www.mirbase.org) were used to infer the potential targeting relationship between miR-219c-5p and FN1. The sequence of the predicted 3′-UTR site where miR-219c-5p binds to FN1 mRNA is shown in Fig. 5a. The dual luciferase reporter assay results showed that miR-219c-5p reduced FN1-WT luciferase activity by 49% (*P* < 0.01) and had no effect on FN1-MUT luciferase activity (Fig. 5a, *P* > 0.05), indicating that miR-219c-5p can target FN1 and affect its synthesis.

### miR-219c-5p targeted FN1 to suppress BSMC proliferation

To observe the effect of miR-219c-5p on BSMC proliferation, we overexpressed or knocked down miR-219c-5p in vitro. The effect of miR-219c-5p on proliferation in BSMCs is shown in Fig. 5B. There was no significant difference in the survival rate of BSMCs between the blank and NC groups (all *P* > 0.05). Compared with that of the blank and NC groups, the viability of BSMCs in the miR-219c-5p group decreased (*P* < 0.05), whereas cell viability in the miR-219c-5p inhibitor group increased (*P* < 0.05). These results indicate that miR-219c-5p can participate in BSMC proliferation by regulating the expression of FN1.

### Effect of miR-219c-5p on the BSMC cycle

**Fig. 6:**
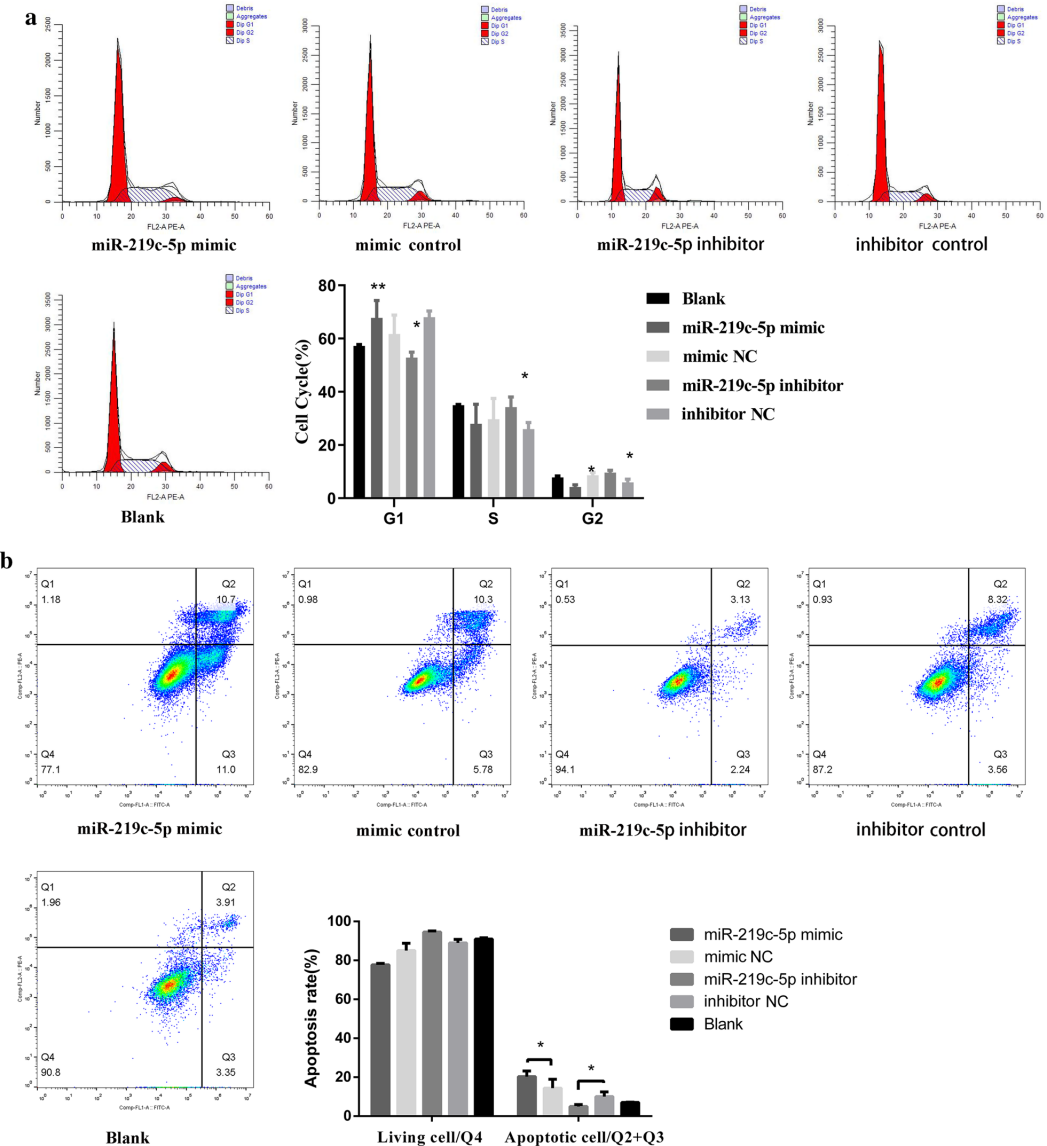
Effect of miR-219c-5p on the cell cycle and apoptosis in BSMCs. **a** Compared with that in the blank and NC groups, the number of BSMCs in the miR-219c-5p group increased in the G1 phase but decreased in the S/G2 phase, while the number of cells in the miR-219c-5p inhibition group decreased in the G1 phase but increased in the S/G2 phase (*P* < 0.05 in all cases). **b** The apoptosis rate of the miR-219c-5p group increased significantly compared with that of the blank and NC groups (*P* < 0.05). However, the apoptosis rate of the miR-219c-5p inhibition group was significantly reduced compared with that of the blank and NC groups (*P* < 0.05)**.** The findings were from three independent experiments. The data are expressed as the means ± SD. *P < 0.05, **P < 0.01 versus mimic NC or inhibitor NC

Compared with that in the blank and NC groups, the number of BSMCs in the miR-219c-5p group increased in the G1 phase but decreased in the S/G2 phase, while the number of cells in the miR-219c-5p inhibition group decreased in the G1 phase but increased in the S/G2 phase (Fig. 6a, *P* < 0.05 in all cases). These data indicate that miR-219c-5p can inhibit the cell cycle by regulating FN1 gene expression and thereby arresting BSMCs in the G1 phase.

### Effect of miR-219c-5p on apoptosis in BSMCs

The apoptosis rate of the miR-219c-5p mock group increased significantly compared with that of the blank and NC groups (*P* < 0.05). However, the apoptosis rate of the miR-219c-5p inhibition group was significantly reduced compared with that of the blank and NC groups (Fig. 6b, *P* < 0.05). These results indicate that miR-219c-5p can promote BSMC apoptosis.

### Effects of miR-219c-5p agomir and antagomir on bladder fibrosis in EAE mice

**Fig. 7:**
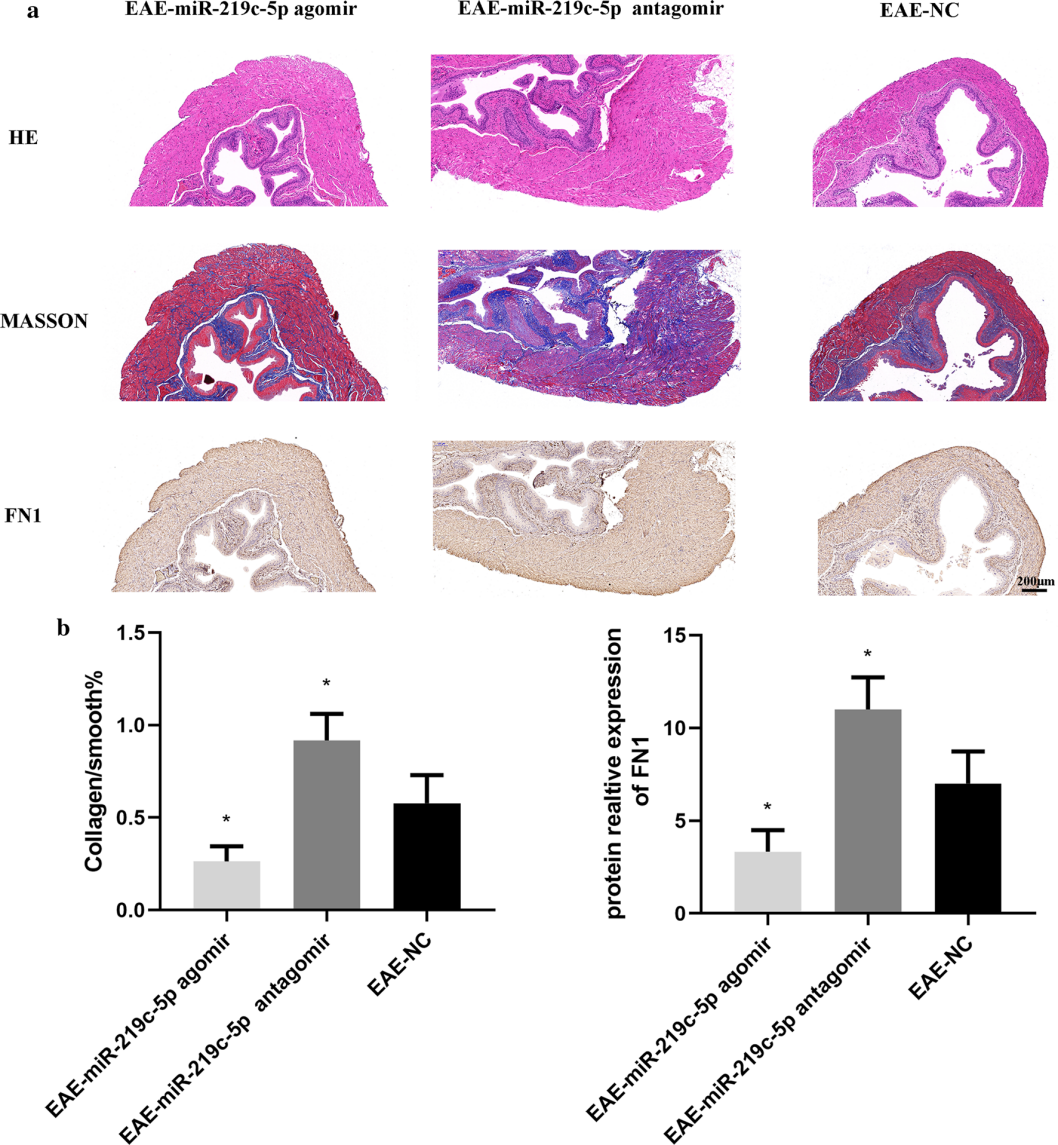
Effects of miR-219c-5p agomir and antagomir on bladder fibrosis in EAE mice. **a** Only the degree of fibrosis and FN1 expression of bladder smooth muscle layer tissue were evaluated. **b** Compared with the EAE-NC group, bladder fibrosis was significantly reduced in the EAE-miR-219c-5p agomir group (*P* < 0.05), and the expression of FN1 content was reduced (*P* < 0.05). The EAE-miR-219c-5p antagomir group had significantly increased bladder fibrosis (*P* < 0.05), and the expression of FN1 increased (*P* < 0.05). The data are expressed as the means ± SD. *P < 0.05 versus EAE-NC

In order to observe the effect of miR-219c-5p on bladder fibrosis in mice EAE model, we overexpressed or knocked down miR-219c-5p in vivo, that is, injection of agomir and antagomir through the tail vein. Compared with the EAE-NC group, bladder fibrosis was significantly reduced in the EAE-miR-219c-5p agomir group (Fig. 7, *P* < 0.05), and the expression of FN1 content was reduced (Fig. 7, *P* < 0.05). The EAE-miR-219c-5p antagomir group had significantly increased bladder fibrosis (Fig. 7, *P* < 0.05), and the expression of FN1 increased (Fig. 7, *P* < 0.05). The above results suggest that miR-219c-5p can participate in the occurrence and development of bladder fibrosis through targeted inhibition of FN1 expression.

## Discussion

In this study, we found that bladder fibrosis in EAE mice was aggravated by clinical symptoms. Moreover, we found that bladder fibrosis severity positively correlated with FN1. Therefore, we speculated that FN1 synthesis could be inhibited by a targeting miRNA, thereby blocking the progression of bladder fibrosis. Two databases, including miRDB (http://mirdb.org/), were used to predict microRNAs that interact with FN1. After comparative analysis, miR-199a-3p, miR-219c-5p and miR-3572-3p were predicted to target FN1. By overexpressing or knocking down miR-199a-3p, miR-219c-5p and miR-3572-3p in bladder smooth muscle in vitro, we found that only miR-219c-5p targeted FN1. A luciferase reporter assay confirmed that miR-219c-5p could inhibit FN1 synthesis. Moreover, CCK8 and flow functional assays confirmed that miR-219c-5p could regulate the function of BSMCs by regulating FN1 synthesis.

Urinary dysfunction, such as frequent urination, urgency, and urinary retention, is a common problem in patients with MS. Treating MS patients with bladder dysfunction remains clinically difficult because there is currently no effective treatment. Altuntas et al. [8] were the first to suggest that the EAE model induced by PEP139-151 is similar to urinary dysfunction in patients with neurogenic bladders. Previous studies have shown that the neurological damage symptoms in EAE mice are associated with prostaglandin E2 E-prostaglandin receptor 3/4 (PGE2 EP3/EP4), providing a new target for neurogenic bladder targeted therapy [26]. Wang et al. [27] reported that human BSMCs proliferation is regulated mainly by extracellular signal-regulated kinase 1/2 activity and by exchange factors directly activated by the cAMP pathway. These findings not only provide new ideas for the treatment of neurogenic bladder but also support our findings.

Fibrosis is described as "wrong body repair", and while its development may involve many mechanisms, myofibroblast activation and the subsequent production of excess extracellular components are essential for fibrosis. Similarly, bladder fibrosis is thought to be involved in BSMC activation and excess extracellular matrix accumulation. Some studies have shown that miRNAs are involved in smooth muscle cell function, including cell proliferation, the cell cycle, and apoptosis [23, 28, 29]. There is growing evidence that many miRNAs play key regulatory roles in bladder fibrosis. Duan et al. [30] demonstrated a novel miR-133 regulatory factor that targets the TGF-β-Smad3 signaling pathway to modulate TGF-β1-induced changes in the BSMC phenotype. A novel antifibrotic function of miR-133 was proposed, which represents a potential target for diagnosis and treatment strategies for bladder fibrosis.

FN1 is a high molecular weight glycoprotein in the extracellular matrix with complex biological functions as a core component. Fibroblasts, vascular endothelial cells, hepatocytes, vascular smooth muscle cells, and other cell types secrete this functional protein, which regulates cell adhesion, proliferation, differentiation, cell morphology maintenance, cell migration promotion, ion exchange, signal transduction, and other functions. Properly increase the expression of FN1 in vitro, the growth of BSMCs increases, but due to the influence of microenvironment (space, nutrition, etc.) in the vivo, when FN1 protein is overexpressed, excessive FN1 protein is deposited in the cytoplasm of the cell, affecting the normal functional activity of the cell causing fibrosis. Numerous studies have reported the regulation of FN1-mediated fibrosis by miRNAs. Gunadi et al. [31] reported that miRNA-206 targeting FN1 is abnormally expressed in multifactorial Hirschsprung's disease and can be used as a potential targeting molecule. Moreover, an interesting phenomenon was found in this study: BSMCs undergo apoptosis after miR-219c-5p is overexpressed. We speculate that BSMC apoptosis may be related to the increase in Bax/Bcl-2 ratio-induced apoptosis after FN1 downregulation [32].

Numerous studies have shown that miRNAs are involved in the tissue fibrosis process. Jing Wu et al. [33] reported that miR-27a can target the AMP-activated protein kinase alpha2 catalytic subunit and activate the TGF-β1 and Smad3 signaling pathways, thereby reducing detrusor fibrosis in streptozotocin-induced diabetic rats. Su-Jin Moon et al. [34] reported that FN1 is an important functional protein in the development of skin fibers. Therefore, we explored whether FN1 is involved in the development of bladder fibrosis and whether miRNAs can target FN1 to regulate bladder fibrosis progression. According to miRBase (http://www.mirbase.org) and miRDB (http://mirdb.org/) predictions, miR-199a-3p, miR-219c-5p and miR-3572-3p may interact with FN1. We overexpressed miR-219c-5p, miR-199a-3p, and miR-3572-3p in vitro and found that FN1 was significantly downregulated when miR-219c-5p was overexpressed. What role might miR-199a-3p and miR-3572-3P play in the development of bladder fibrosis? Ying Tao [35] et al. reported that miR-199a-3p promotes cardiomyocyte proliferation by inhibiting Cd151 expression. We speculated through miRDB (http://mirdb.org/) that miR-3572-3P may inhibit cell proliferation by targeting cyclin-dependent kinase 6. Combining the results of in vivo and in vitro experiments, only miR-219c-5p can better regulate FN1 and participate in bladder fibrosis.

## Conclusions

Overall, this study reported that miR-219c-5p is better at targeting FN1 than miR-199a-3p and miR-3572-3p. This study demonstrated that miR-219c-5p blocks bladder fibrosis by inhibiting FN1 expression.

In conclusion, this study confirmed that FN1 and miR-219c-5p are associated with bladder fibrosis in MS mice. We also show that miR-219c-5p can regulate bladder fibrosis by targeting FN1. A novel antifibrotic function of miR-219c-5p is proposed, which may represent a potential target for the diagnosis and treatment of bladder fibrosis.

## Data Availability

The datasets used during the current study available from the corresponding author on request.

## Abbreviations

MS: Multiple sclerosis
EAE: Experimental autoimmune encephalomyelitis
BSMCs: Bladder smooth muscle cells
ECM: Extracellular matrix
FBS: Fetal bovine serum fetal bovine serum
FN1: Fibronectin 1
qPCR: Quantitative real-time PCR
α-SMA: α-Smooth muscle actin
CS: Clinical score
